# Tag Thy Neighbour: Nanometre-Scale Insights Into Kinetoplastid Parasites With Proximity Dependent Biotinylation

**DOI:** 10.3389/fcimb.2022.894213

**Published:** 2022-05-06

**Authors:** Vincent Geoghegan, Jeremy C. Mottram, Nathaniel G. Jones

**Affiliations:** Department of Biology, York Biomedical Research Institute, University of York, York, United Kingdom

**Keywords:** BioID proximity labeling, leishmania, trypanosoma, biotinylation, kinetoplastid, proteomics, mass spectrometry - LC-MS/MS, APEX2, BirA enzyme

## Abstract

Proximity labelling is a powerful and rapidly developing technology for exploring the interaction space and molecular environment of a protein of interest at the nanometre scale. In proximity labelling, a promiscuous biotinylating enzyme is genetically fused to the protein of interest, initiation of labelling then results in the biotinylating enzyme generating reactive biotin which covalently ‘tags’ nearby molecules. Importantly, this labelling takes place *in vivo* whilst the protein of interest continues to perform its normal functions in the cell. Due to its unique advantageous characteristics, proximity labelling is driving discoveries in an ever increasing range of organisms. Here, we highlight the applications of proximity labelling to the study of kinetoplastids, a group of eukaryotic protozoa that includes trypanosomes and *Leishmania* which can cause serious disease in humans and livestock. We first provide a general overview of the proximity labelling experimental workflow including key labelling enzymes used, proper experimental design with appropriate controls and robust statistical analysis to maximise the amount of reliable spatial information that is generated. We discuss studies employing proximity labelling in kinetoplastid parasites to illustrate how these key principles of experimental design are applied. Finally, we highlight emerging trends in the development of proximity labelling methodology.

## Introduction

Discovery of interaction partners is often a key step towards understanding the biological role of a protein of interest. A common approach is affinity purification of the protein of interest followed by identification of co-purifying proteins by mass spectrometry (AP-MS). AP-MS is a well-established technique that can yield valuable insights (Gingras et al., 2007). However, the success of an AP-MS experiment depends on finding lysis conditions that are sufficient to solubilise the protein of interest (bait), but not so stringent that associations with interactors (prey) are disrupted. Preserving interactions therefore necessitates relatively gentle lysis and washing conditions, unfortunately this can be permissive to formation of post-lysis interactions which do not occur *in vivo* (Branon et al., 2017). Proximity labelling is a new paradigm for exploring interactions involving a protein of interest which bypasses the requirement to preserve interactions during lysis and enrichment. This key advantage of proximity labelling lies in the fact that the protein of interest is expressed as a fusion to an enzyme that generates reactive biotin molecules which then covalently tag nearby proteins *in vivo* (Samavarchi-Tehrani et al., 2020). Interacting proteins will continue to associate and dissociate during the labelling period, potentially leading to an accumulation of biotinylation signal over time, in contrast to AP-MS which can only capture interactors at the point of lysis. Two-hybrid interaction assays such as yeast two-hybrid, like proximity labelling, can detect *in vivo* interactions and potentially offer a very detailed view of interaction networks by virtue of the pairwise screening for bait-prey interactions. In two-hybrid assays, direct interaction partners can be discovered in a high-throughput manner, although the set up costs can be quite high and proteins are often expressed outside of their normal context (Mehla et al., 2017).

**Figure 1:**
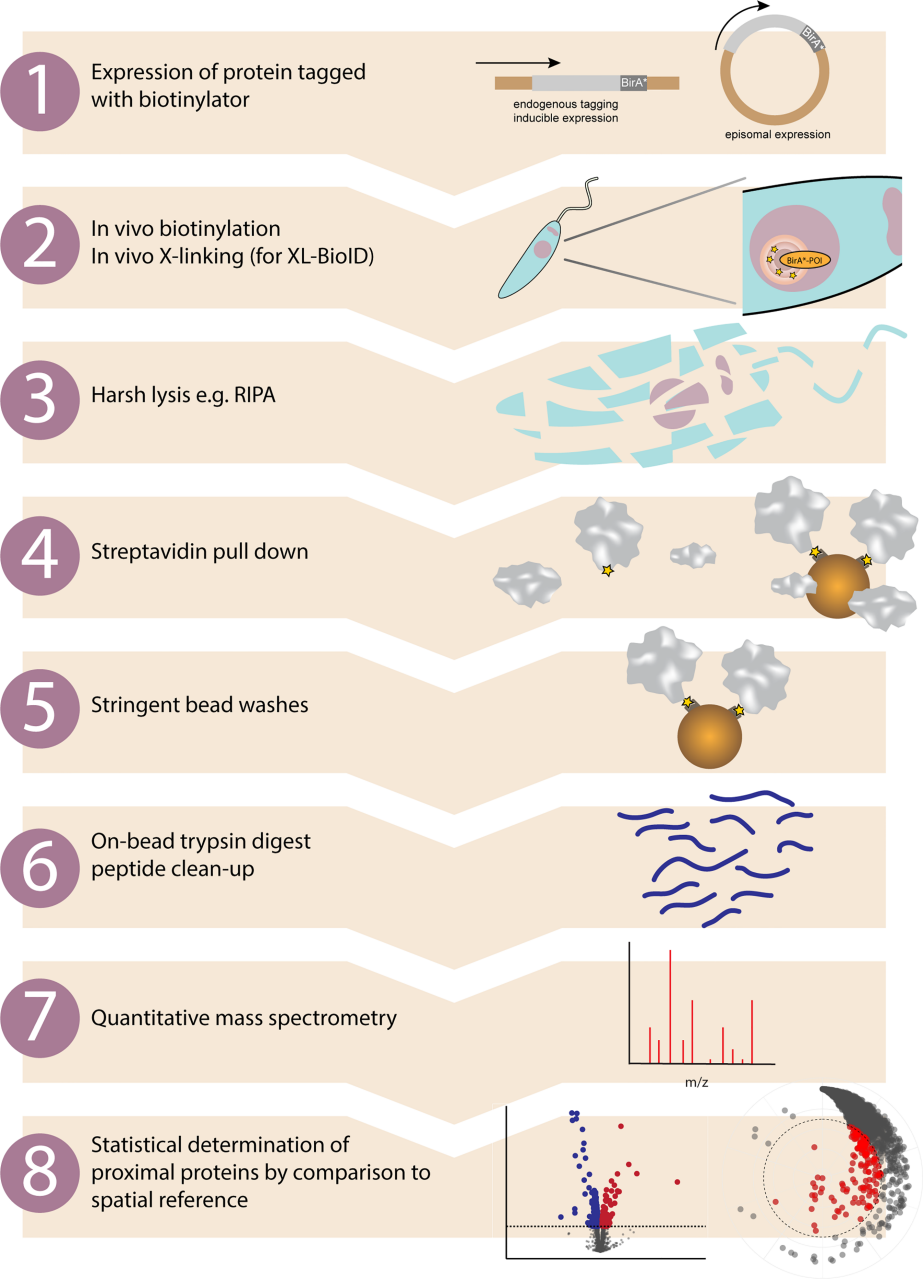
Proximity biotinylation workflow in kinetoplastids. Overview of the main experimental steps involved in a proximity biotinylation experiment. 1. Protein of interest is either tagged at the endogenous locus with a biotinylator such as BirA* or APEX2, or a tagged version is expressed from a different locus. 2. Adding exogenous biotin to the growth medium (BirA*) or biotin-phenol then H_2_O_2_ (APEX2) initiates *in vivo* proximity biotinylation. If performing XL-BioID, this may then be followed by an *in vivo* cross-linking step to increase capture of proximal proteins. A nuclear protein fused to BirA* is shown as an example. 3. The major advantage of proximity biotinylation is being able to completely lyse and solubilise cells with harsh lysis conditions such as radioimmunoprecipitation assay (RIPA) buffer. 4. Proximal proteins covalently tagged with biotin are enriched by incubating lysate with streptavidin beads. 5. Non-proximal proteins are washed off with a series of stringent bead washes. 6. Proteins remaining on beads are digested with trypsin and released peptides are desalted using C18. 7. Peptides are identified and quantified by quantitative mass spectrometry. Quantitation may be label free, TMT or SILAC based. 8. Enrichment of identified proteins vs. a control sample is calculated and statistical testing performed, e.g. with SAINTq or limma. Proteins significantly enriched in the sample vs. the spatial reference sample are classed as proximal proteins.

Proximity labelling is a broad term collecting together a range of approaches which can be used to characterise proximal molecular space not just for proteins, but other biomolecules such as DNA and RNA. Since the majority of proteomic studies use a bait fused to an enzyme which generates reactive biotin to carry out proximity dependent biotinylation, we focus on this subset of proximity labelling here. An overview of the complete proximity biotinylation workflow is shown in **Figure 1**.

**Figure 2:**
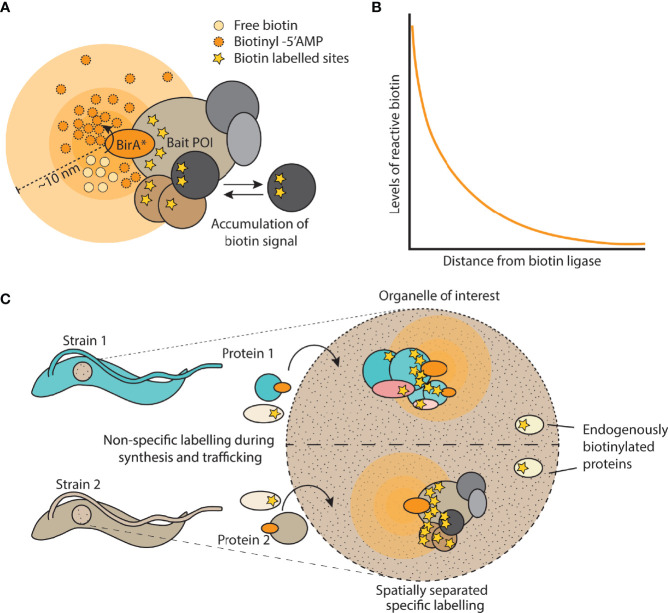
Overview of proximity biotinylation by BirA*. **(A)** Cartoon depicting a bait protein of interest (POI) fused to BirA*. Free biotin is converted into a reactive biotinyl-5’AMP which reacts with lysine residues in proteins within a 10 nm labelling radius. Signal accumulates over time due to dynamic changes in the protein complex. **(B)** Depiction of the exponential decay of reactive biotin levels as distance increases from the biotin ligase. **(C)** Example of using spatial or organellar control strains, in which a different protein is tagged with the biotin ligase and compared against the strain expressing the control protein. This allows for the computational removal of endogenously biotinylated proteins as well as labelled proteins that are not specific to complexes of interest.

The concept of proximity dependent biotinylation was proposed by Choi-Ree et al. based on information gained from prior fundamental studies on the structure and function of *E. coli* biotin ligase (Choi-Rhee et al., 2004). *E. coli* BirA biotin ligase specifically biotinylates a single lysine in biotin carboxyl carrier protein in a two step reaction involving a reactive intermediate that is normally held in the active site. However, previous work identified two mutant forms of BirA, G115S and R118G which have reduced affinity for the biotinylation reaction intermediate biotin adenylate (biotinyl-5’-AMP) compared to the wild-type enzyme (Kwon and Beckett, 2000). Since biotinyl-5’-AMP is a reactive and short lived biotin adenylate, this led Choi-Ree et al. to propose that “*these mutant proteins might leak bio-5′-AMP into the solvent where it would act as a promiscuous chemical biotinylation reagent in a proximity-dependent manner*” (Choi-Rhee et al., 2004) (**Figure 2A**). Indeed, *in vitro* experiments were performed demonstrating proximity dependent biotinylation using the R118G promiscuous biotin ligase variant, BirA*. *In vivo* experiments were also performed showing the promiscuous nature of biotinylation, but targets were not identified. The first demonstration of proximity dependent biotinylation *in vivo* arrived much later in a study by Roux et al. who fused BirA* (R118G) to lamin-A and demonstrated enrichment of proximal and interacting proteins, naming the technique BioID (Roux et al., 2012).

Several excellent general overviews of proximity biotinylation have been written which should be consulted when planning experiments (Samavarchi-Tehrani et al., 2020; Qin et al., 2021a). Here, we provide an overview of the technique and focus on its practical application to research on kinetoplastids.

## Biotin Ligases and Peroxidases

There are two main classes of enzyme used for proximity dependent biotinylation, promiscuous biotin ligases and peroxidases (Samavarchi-Tehrani et al., 2020). The original *E. coli* R118G biotin ligase mutant has been widely used in over 300 studies and remains an important tool. Labelling kinetics of BirA* are relatively slow, requiring 18-24 hr incubations with exogenous biotin to achieve optimal labelling, which does not allow studies of more dynamic processes that occur in seconds to minutes. However, slow kinetics may help to reduce background as only true interactors will dwell long enough to become biotinylated, which may explain the success of BirA* for characterising stable protein complexes (Gingras et al., 2019). Although *in vivo* labelling by BirA* is relatively slow, it is worth noting that first permeabilising cells and performing *‘in vitro* BioID’ enables labelling in 10-15mins (Remnant et al., 2019). This suggests that BirA* can perform rapid labelling under certain conditions, in this case possibly due to effects of detergents or the removal of quenchers. Further investigation of factors influencing promiscuous biotinylation is therefore required, as our understanding of the mechanism is incomplete.

More recent engineered biotin ligases have been developed such as BioID2, BASU, AirID, ultraID, miniTurbo and TurboID (Kim et al., 2016; Branon et al., 2018; Ramanathan et al., 2018; Kido et al., 2020; Zhao et al., 2021). These enzymes show a spectrum of labelling kinetics with BioID2 having modest improvements in labelling efficiency over BirA*, whilst AirID enables faster labelling in ~3hrs. TurboID and miniTurbo were created by directed evolution of BirA* and rapidly biotinylate proximal proteins in 10-15mins (Branon et al., 2018). Because activity screening for these variants was carried out at the growth temperature of yeast, 26°C, they may be well suited for use in organisms that are grown at lower temperatures such as *Leishmania* promastigotes. They have already been used in a number of studies, however they each have limitations. TurboID, which is the more efficient enzyme of the two, also has a higher affinity for biotin and therefore carries out extensive biotinylation using the low levels of biotin already present in culture media. This prevents temporal control of biotinylation, thus decreasing spatial resolution and increasing background, because the enzyme is constitutively biotinylating proximal proteins as soon as the transgene is expressed. MiniTurbo labels more slowly but has less background activity in the absence of exogenous biotin, enabling better temporal control of labelling (Branon et al., 2018). The DNA binding domain present in BirA was deleted to create miniTurbo which may help to reduce background labelling of chromatin associated proteins, however this appears to have destabilised the protein in some contexts (May et al., 2020). BASU, an engineered biotin ligase from *Bacillus subtilis* also lacks a DNA binding domain and was presented as enabling proximity labelling in a timescale of 1min, however a later study did not find it was significantly faster than BirA* (Branon et al., 2018). Most recently, ultraID has been developed by directed evolution of biotin ligase from the thermophilic bacterium *Aquifex aeolicus* and appears to be a very promising enzyme, combining fast labelling kinetics (10mins), small size (20kDa) and low background activity in the absence of exogenous biotin (Zhao et al., 2021). The directed evolution study leading to ultraID showed that just two mutations in the biotin binding site generated a highly active promiscuous biotin ligase, contrasting with the directed evolution of TurboID which has a total of 15 mutations compared to the wild-type enzyme. Aside from the R118S mutation, the mutations in TurboID appear to be spread throughout the structure of the biotin ligase, exactly how they contribute to augmenting promiscuous biotinylation activity is unclear, further highlighting the need for fundamental studies on the mechanism of proximity dependent biotinylating enzymes.

The focus of biotin ligase engineering efforts has been to produce enzymes with faster labelling kinetics. This is evidently required for studies of dynamic cellular processes, but faster is not necessarily better for every application. A side by side comparison of nuclear pore proteins Nup43 and Nup53 fused to BirA* or TurboID in mammalian cells, showed that coverage of the nuclear pore complex was similar for both biotinylating enzymes but that the total number of proteins identified in the TurboID samples was ~2.7 fold higher, the additional hits comprising proteins present in other subcellular compartments such as the ER and mitochondria (May et al., 2020). This indicates the higher activity of TurboID may lead to a higher background due to off target labelling of bystander proteins, whereas the slower labelling of BirA* may actually lead to a cleaner background because only true interactors reside long enough within the labelling radius to become biotinylated.

Peroxidase based proximity biotinylation proceeds *via* a different mechanism compared to biotin ligase based approaches. Cells expressing the peroxidase fused to a protein of interest are first incubated with a phenol derivative of biotin such as biotin-phenol. Proximity biotinylation is then initiated with the addition of H_2_O_2_. Peroxidases such as APEX2 catalyse H_2_O_2_ mediated oxidation of biotin-phenol, generating highly reactive phenoxyl-biotin radicals which then covalently tag electron rich amino acid side chains, such as tyrosine, in proximal proteins (Lam et al., 2015). A key advantage of APEX2 catalysed proximity biotinylation is that it is very rapid, permitting labelling times as short as 1 min to be used which has allowed dynamic processes such as G-protein coupled receptor signalling to be followed (Lobingier et al., 2017; Paek et al., 2017). If the protein of interest is a cell surface protein, horseradish peroxidase (HRP) can be used to biotinylate proximal proteins either by expressing the protein fused to HRP (Miyagawa-Yamaguchi et al., 2014), or targeting the protein with a specific HRP-conjugated antibody (Bar et al., 2018). HRP has a higher activity than APEX2, unfortunately it is not active in the cytosol which limits its application to extracellular facing proteins (Hopkins et al., 2000). While being well suited to taking rapid snapshots of proximal molecular environments, peroxidase based labelling has its own limitations. Labelling is initiated by H_2_O_2_ which is toxic and can lead to membrane disruption, thus it may be less suitable for whole organism proximity biotinylation studies, Finally, as well as the expressed peroxidase fusion protein, endogenous peroxidases may also catalyse biotinylation and contribute to the background signal.

Cellular compartments such as the nucleus or endoplasmic reticulum (ER) each have their own distinct chemical micro-environments that can strongly influence the activity of biotinylating enzymes. As part of their thorough characterization of evolved BirA variants, Branon et al. compared the activity of BirA*, TurboID and miniTurbo in the cytosol, nucleus, ER lumen, ER membrane and mitochondrial matrix of HEK293T cells (Branon et al., 2018). They observed that all 3 ligases had lower activity in the ER lumen and mitochondrial matrix compared to the other compartments. Comparing between the three ligase variants, BirA* generated a relatively consistent biotinylation signal across all compartments, whilst the activity of TurboID was quite variable, being lowest in the nucleus, but higher in the ER lumen and mitochondrial matrix, relative to BirA*. In the cytosol, TurboID and miniTurbo had highest activity relative to BirA*, compared to the other organelles.

## The Labelling Radius

A key question often raised when discussing proximity biotinylation techniques is the labelling radius. The first estimation of the *in vivo* labelling radius used the Y-complex, a sub-complex of the nuclear pore complex, as a molecular ruler to measure the labelling distance (Kim et al., 2014). BirA* fusions were created for 5 members of the Y-complex which has known architecture and dimensions. Analysis of the detected abundance of co-enriched subunits indicated the labelling radius of BirA* was ~10nm. In a separate study, BioID was performed in cells expressing BirA* fused to a centromeric histone variant (Remnant et al., 2019). Deposition of biotin along chromatin was directly visualised by electron microscopy which revealed a narrow distribution either side of CENP-A BirA* along the linear chromatin, with a radius of ~10nm, in agreement with the study on the Y-complex. For context, an averaged sized protein of 50kDa has a diameter of ~5nm assuming a spherical shape (Erickson, 2009), therefore proximity biotinylation has a high spatial resolution; for investigations of large macromolecular complexes multiple subunits may need to be fused to a biotinylator or a cross-linking step included to improve coverage (Geoghegan et al., 2021). However, as has been previously highlighted, it is more accurate to view the labelling radius as a contour map, not having a hard proximity cutoff, but rather a decreasing probability of labelling with distance (Qin et al., 2021a). The exception to this are cases when the biotinylator is close to a membrane which does act as a hard cutoff, being impermeable to reactive biotin species.

It is also important to consider the nature of the concentration gradient of reactive biotin molecules formed from the source. A gradient will form as a result of diffusion away from the source but also due to quenching/breakdown of the reactive biotin molecules over time. Both these processes are expected to combine to create an *exponentially* decreasing concentration of reactive biotin (**Figure 2B**) as a function of distance from the biotinylator which would contribute to the high spatial resolution (Wartlick et al., 2009). Thus biotinylation of a complex will drop off sharply with distance from the bait, direct interactors will be strongly labelled, but indirect interactors much less so, this is indeed what is observed from proximity biotinylation data. On this basis, it is tempting to infer distance of a proximal protein from the bait by analysing its relative amounts as quantified by mass spectrometry in the enriched biotinylated material. However, there are too many factors other than distance which determine the extent of proximity biotinylation, such as availability of lysine residues, residence time in the complex, orientation of the protein and copy number of the protein.

Measurements of the labelling radius of proximity biotinylation using BirA* indicate a high spatial resolution of ~10nm, however the labelling radius will be determined in each case by the half-life of the reactive intermediate involved and the subcellular environment. Also, measurements of labelling radius are based on stable, long lived structures. In reality, there will be many dynamic processes taking place in the vicinity, proteins may diffuse into the labelling radius and become biotinylated without being part of the structure. Additionally, for conventionally designed studies, the labelling radius is active throughout the entire lifetime of the protein, from its point of synthesis to the point of degradation. This can make the resulting datasets quite complex and could be a particular issue for the newer generation of rapid biotin ligases which may be more prone to tagging ‘bystander’ proteins. An excellent control for off-target biotinylation is a spatial reference, where a cell line is generated expressing the biotinylator localised to the same sub-cellular region as the protein of interest. The control cell line could even express a protein of potential interest tagged with a biotinylator. In this scenario, the two tagged baits act as reciprocal controls (**Figure 2C** and see experimental design section).

## Proximity Biotinylation in Kinetoplastids

Kinetoplastids are a large group of flagellated protozoa named after the kinetoplast, a characteristic mass of DNA contained in the single mitochondrion (Povelones, 2014). Some kinetoplastids exist as free-living organisms, whilst others require a host to survive and can infect a range of organisms such as plants, insects or animals (Kostygov et al., 2021). Two genera of kinetoplastids cause serious diseases in humans and livestock: *Leishmania* causing the leishmaniases and *Trypanosoma* causing sleeping sickness and Chagas disease (Burza et al., 2018; Libisch et al., 2021; Pays and Nolan, 2021). As well as for their impact on human health, kinetoplastids are also studied for their rather unique way among eukaryotes of carrying out molecular processes, such as absence of introns, constitutive transcription of genes, widespread trans-splicing and highly divergent or even unique protein complexes of fundamental importance to cell biology, such as the kinetochore or pre-replication complex (Tiengwe et al., 2012; Akiyoshi and Gull, 2014; Clayton, 2016; De Pablos et al., 2016). Comprehensive molecular toolkits allowing functional genetics make these organisms powerful non-opisthokont model organisms.

A number of studies (**Table 1**) have made use of proximity biotinylation to investigate protein complexes in different sub-cellular compartments of kinetoplastids, with a notable focus on characterising cytoskeletal associated complexes, for which proximity biotinylation is well suited. We highlight selected studies that illustrate the range of technical approaches used in kinetoplastids.

**Table 1:** List of proximity biotinylation studies conducted in kinetoplastids.

Bait Protein	Species	Gene ID	Bait Expression	Control	Enzyme/method	Reference
MORN1	*T. brucei* Procyclic Forms	*Tb927.6.4670*	Tet Inducible Allele	Plus/Minus Tet	BirA*/BioID	(Morriswood et al., 2013)
PLK	*T. brucei* Procyclic Forms	*Tb927.7.6310*	Tet Inducible Allele	Plus/Minus Tet	BirA*/BioID	(McAllaster et al., 2015)
SAS-4	*T. brucei* Procyclic Forms	*Tb927.11.3300*	Tet Inducible Allele	Plus/Minus Tet	BirA*/BioID	(Hu et al., 2015)
CIF1	*T. brucei* Procyclic Forms	*Tb927.11.15800*	Tet Inducible Allele	Plus/Minus Tet	BirA*/BioID	(Zhou et al., 2016)
TbSAS-6, TbCEP57, TbPOC11	*T. brucei* Procyclic Forms	*Tb927.9.10550, Tb927.10.5990, Tb927.11.6560*	Tet Inducible Allele	Plus/Minus Tet	BirA*/BioID	(Dang et al., 2017)
RBP9, RBP10	*T. brucei* Procyclic Forms	*Tb927.11.12120, Tb927.8.2780*	Tet Inducible Allele	Strain Expressing GFP-BirA*	BirA*/BioID	(De Pablos et al., 2017)
NuSAP1, NuSAP2, Kif13-1, Mlp2, AUK1	*T. brucei* Procyclic Forms	*Tb927.11.8370, Tb927.9.6110, Tb927.9.3650, Tb927.9.1340, Tb927.11.8220*	Tet Inducible Allele	Plus/Minus Tet	BirA*/BioID	(Zhou et al., 2018b)
TOEFAZ1 (=CIF1)	*T. brucei* Procyclic Forms	*Tb927.11.15800*	Tet Inducible Allele	Plus/Minus Tet	BirA*/BioID	(Hilton et al., 2018)
CIF1	*T. brucei* Procyclic Forms	*Tb927.11.15800*	Tet Inducible Allele	Comparison to previous datasets	BirA*/BioID	(Zhou et al., 2018a)
FPRC	*T. brucei* Procyclic Forms	*Tb927.10.6360*	Tet Inducible Allele	Comparison to previous datasets	BirA*/BioID	(Hu et al., 2019)
ZapE1, ZapE2, IscU, LigK-beta	*T. brucei* Procyclic Forms	*Tb927.7.6930, Tb927.10.8070, Tb927.9.11720, Tb927.7.600*	Endogenous Tagging	Reciprocal organellar spatial controls	BioID2	(Pyrih et al., 2020)
FAZ27	*T. brucei* Procyclic Forms	*Tb927.9.8350*	Tet Inducible Allele	Plus/Minus Tet	BirA*/BioID	(An et al., 2020)
Spef1	*T. brucei* Procyclic Forms	*Tb927.4.3130*	Endogenous Tagging	Wild Type Cells	BirA*/BioID	(Dong et al., 2020)
Unc119	*T. brucei* Procyclic Forms	*Tb927.2.4580*	Tet Inducible Alleles	Overlapping hits from N- and C-terminal tagged experiments	BioID2	(Pandey et al., 2020)
TAC102	*T. brucei* Procyclic Forms	*Tb927.7.2390*	Tet Inducible Allele	Plus/Minus Tet	BirA*/BioID	(Baudouin et al., 2020)
KHARON	*L. mexicana* Promastigotes	*LmxM.36.5850*	Episomal Allele	Wild Type Cells	BirA*/BioID	(Kelly et al., 2020)
DRC1, AC1, FS179, AC1Δ45	*T. brucei* Procyclic Forms	*Tb927.10.7880, Tb927.11.17040, Tb927.10.2880*	Endogenous Tagging	DRC1 vs WT, AC1/FS179 vs WT and AC1Δ45(cytosolic control)	APEX2	(Vélez-Ramírez et al., 2021)
KKT2, KKT3, KKT19	*L. mexicana* Promastigotes	*LmxM.36.5350, LmxM.34.4050, LmxM.09.0410*	Endogenous Tagging	Reciprocal organellar spatial controls	BirA*/miniTurbo/XL-BioID	(Geoghegan et al., 2021)
BDF5	*L. mexicana* Promastigotes	*LmxM.09.1260*	Endogenous Tagging	Reciprocal organellar spatial controls	BirA*/miniTurbo/XL-BioID	(Jones et al., 2021)

### Trypanosoma brucei

**Figure 3:**
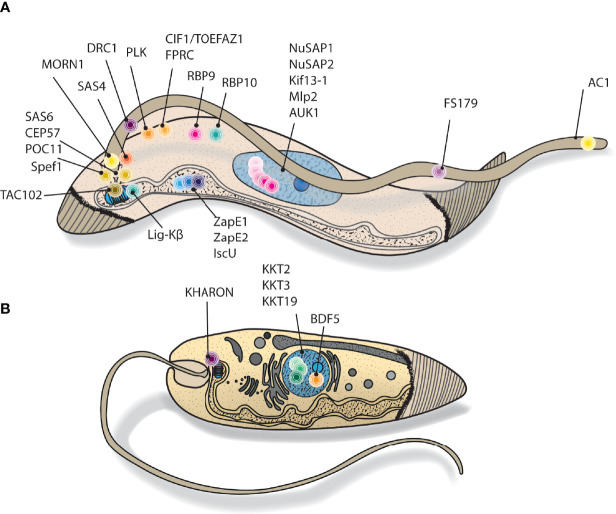
Cartoon depiction of kinetoplastid targets explored using proximity biotinylation. Cutaway style drawing, showing stylised cellular location of **(A)** *T. brucei* and **(B)** *Leishmania* proximity biotinylation bait proteins. N.B. many of the represented proteins have dynamic localisations throughout the cell cycle.

The majority of proximity biotinylation studies in kinetoplastids to date have investigated protein complexes in procyclic form *Trypanosoma brucei* (**Figure 3A**). This is likely because relative to other kinetoplastids, a well-developed genetic toolbox in *T. brucei* has been available for longer, making it straightforward to endogenously tag a protein of interest with a promiscuous biotin ligase or overexpress a bait protein fused to a biotin ligase. Morriswood et al. reported the first successful use of proximity biotinylation in kinetoplastids in 2013, in fact this was only the second reported use of proximity biotinylation to probe *in vivo* complexes after the original Roux et al. study (Morriswood et al., 2013). The authors investigated a discrete cytoskeletal structure known as the bilobe, which localises to just above the flagellar pocket collar. To interrogate the composition of the bilobe, a known marker, MORN1, was N-terminally tagged with BirA* and this construct was expressed from the ribosomal locus under the control of the tetracycline inducible promoter, giving modest overexpression compared to endogenous levels. Correct expression and localization of the BirA*-MORN1 was first confirmed by western blotting and fluorescence microscopy. Biotinylation of proximal proteins was carried out for 24hrs, before insoluble cytoskeletal proteins were extracted to provide some enrichment for the complex of interest. Biotinylated material was then purified from SDS solubilised cytoskeletal extract. Three biological replicates were performed, no tetracycline induction of BirA*-MORN1 expression served as the control group. Protein hits were inspected manually to determine candidate proximals, those that had a high number of identified peptides in the induced vs. non-induced samples and with an uncharacterised localisation were selected for screening by fluorescence microscopy. Of the 10 screened proteins, 7 were found to be novel components of the bilobe structure, demonstrating the power of proximity biotinylation for discovering cytoskeletal protein complexes that are otherwise biochemically challenging to profile due to their low solubility.

Subsequent applications of proximity biotinylation in *T. brucei* have mostly been very similar in experimental design. The majority of studies relied on wild-type parasites or uninduced parasites as negative controls, rather than using spatial references to estimate off target biotinylation. The exception to this is work by De Pablos et al. and Pyrih et al., where compartment matched spatial references were used to more accurately estimate off target biotinylation (De Pablos et al., 2017; Pyrih et al., 2020). For example, Pyrih et al. investigated 4 mitochondrial bait proteins by proximity biotinylation, using the baits as reciprocal controls.

Overall, 13 of the 16 studies used BirA* tagged proteins and these were mainly overexpressed under the control of a tetracycline inducible promoter. Overexpression has the potential for increasing off-target biotinylation, but this is mitigated by adjusting expression levels of the fusion protein with tetracycline to approximately match levels of the endogenous protein. Dong et al. endogenously tagged Spef1 with BirA* to characterise microtubule quartet associated proteins residing in the region of the flagellum between the bilobe and basal body (Dong et al., 2020). Correct localization of the fusion protein was verified by microscopy and spatially focused biotinylation was demonstrated by incubating parasites with biotin, followed by visualization of proximity biotinylation with fluorescent streptavidin. To maximise capture of proximal proteins, separate experiments were performed with both N and C-terminally BirA* tagged Spef1. As with previous studies examining cytoskeletal complexes, a pre-enrichment step was carried out by isolating the cytoskeleton which remains in the insoluble fraction following lysis with a gentle detergent such as NP-40. Unusually for this type of study, an estimated absolute amount was calculated for each identified protein based on the exponentially modified protein abundance index (emPAI) (Ishihama et al., 2005). Putative proximals present in high absolute amounts and absent in the control samples were selected for follow up validation and functional studies. Here control samples were wild-type parasites. All 8 of the uncharacterised proximal proteins followed up were found to either co-localise with Spef1 or to nearby flagellar associated structures. One of these, SAF1, was shown to be a novel component of the basal body and required for correct spatial organization of structures at the base of the flagellum.

Another example of exploring proximal proteins by using both N and C terminal BirA*-bait fusions are two studies by Zhou et al., in which CIF1 proximal proteins were identified (Zhou et al., 2016; Zhou et al., 2018a). CIF1 localises to the tip of the flagellum attachment zone and has an essential role in cytokinesis initiation. CIF1-BirA* was overexpressed in the first study and proximity biotinylation identified 160 potential proximal proteins, although this number appears to be based on simple manual inspection of identified proteins. Twelve of these proteins were imaged by fluorescence microscopy, 4 of which were found to be close to CIF1, one of these was named CIF2 and association with CIF1 was confirmed by co-immunoprecipitation. A third member of the putative complex, CIF3 was discovered in separate work through a tagging and fluorescence microscopy screen. Because CIF3 was not in the initial proximity biotinylation screen using a C-terminally tagged bait, further work was carried out using N-terminally BirA* tagged CIF1 as a bait. This second proximity biotinylation analysis of CIF1 identified more than 500 proteins in total, proteins deemed to be common contaminants were manually removed from the dataset leaving 305 putative proximals. Fluorescence microscopy localisation screening reduced this down to 52 proteins confirmed as proximal to CIF1, 33 of which were detected in both the BirA*-CIF1 and CIF1-BirA* datasets, showing good, but not complete overlap between datasets generated from the different tagged termini.

There are few reports comparing proximity biotinylation with other approaches commonly used for identifying interaction partners. Baudouin et al. compared the performance of proximity biotinylation, immunoprecipitation and yeast two-hybrid assay in *T. brucei* for detecting proteins interacting with TAC102, a mitochondrial component of the tripartite attachment complex (TAC) which links the basal body of the flagellum to mitochondrial DNA (kDNA) (Baudouin et al., 2020). Proteins are usually targeted to the mitochondrion by an N-terminal targeting signal, which would normally preclude fusing the BirA* ligase here, however in the case of TAC102 the mitochondrial targeting signal is actually located at the C-terminus, therefore BirA* was fused to the N-terminus and correct mitochondrial import of the fusion protein was observed, an excellent example of where correct placement of the BirA* ligase on the bait was critical to the success of the project. In this work, 4 biological replicates were used and enrichment was calculated relative to uninduced control samples. To define proximal proteins, a t-test was performed, proteins enriched at least 3-fold and with significance p <=0.01 were considered proximal, giving a final list of 77 proteins, 47 of these were predicted to be mitochondrial. From the immunoprecipitation experiment of native untagged TAC102, 100 proteins were found significantly enriched, 49 of which were predicted to be mitochondrial. Only 9 proteins were detected as significantly enriched in both proximity biotinylation and immunoprecipitation approaches, of which 3 localised to the kDNA/TAC region. The yeast two-hybrid screen identified a known TAC component, p166, but otherwise generated few confident hits.

In this comparison, results from proximity biotinylation were somewhat disappointing because using BirA*-TAC102 as bait did not lead to detection of any other known TAC proteins. Immunoprecipitation results were slightly better, identifying one known TAC component, p166. The two approaches did identify 3 potential novel TAC components, but the low recall of known complex members suggests that TAC presents distinct challenges and further optimisation is required. Biotinylation was carried out over 6hrs, so one possible improvement could be extending the duration of biotinylation.

In order to capture snapshots of the local protein environment with higher temporal resolution, proximity biotinylating methods using enzymes with faster kinetics need to be established in kinetoplastids. To this end, Velez-Ramirez et al. reported the first use of APEX2 in kinetoplastids, using APEX2 fused to flagellar proteins to explore flagellum subdomains in *T. brucei* (Vélez-Ramírez et al., 2021). Three flagellar localised baits were used as spatial probes, DRC1, AC1 and FS179, all fused with APEX2 at their C-terminus by endogenous tagging. DRC1 is present at the axoneme, an arrangement of microtubules forming the core structure of the flagellum, AC1 localises to the tip of the flagellum and FS179 localises to the flagellum attachment zone and is excluded from the tip. Procyclic parasites were pre-incubated with biotin-phenol for 1hr, then labelling was carried out for 1min by treatment with H_2_O_2_, followed by a quenching step. Parasites were lysed under relatively gentle conditions so that the lysate could be fractionated into soluble and non-soluble parts. The non-soluble fraction containing the majority of the cytoskeletal proteins was later solubilised in SDS for capture of biotinylated material. Very stringent and extensive streptavidin bead washing conditions were used in this workflow, for example buffers containing 8M urea, taking full advantage of the strong biotin-streptavidin interaction to strip the beads of non-biotinylated proteins. To define a proximal proteome for the baits, a semi-quantitative strategy was used based on spectral counting. For the bait AC1, known flagellum tip proteins in the dataset were used to set enrichment thresholds, proteins enriched above this threshold relative to samples from wild-type parasites were considered proximal. The AC1 proximity proteome, at 48 proteins, was more focussed than the 697 proteins in the DRC1 proximity proteome and 400 in the FS179 proximity proteome, consistent with more spatially restricted labelling. Interestingly, proteins with possible roles in signal transduction at the flagellum tip, such as adenylate cyclases, were detected, pointing towards future applications of the methodology in kinetoplastids to examine much more dynamic processes than flagellum structure.

### Leishmania mexicana

Proximity biotinylation has only relatively recently been applied to study *Leishmania* cell biology with a few examples reported to date (**Figure 3B**). Kelly et al. were the first to report the use of proximity biotinylation in *Leishmania*, using the approach to explore proteins proximal to KHARON (KH) (Kelly et al., 2020). KH is a cytoskeletal protein important for replication of the intracellular amastigote stage with multiple localisations: at the base of the flagellum, the subpellicular microtubules around the cytosol and mitotic spindles. To discover KH proximal proteins that may be important for function at the subcellular regions, BirA*-KH was episomally overexpressed and biotinylation carried out overnight. The cytoskeletal fraction was purified for subsequent streptavidin enrichment of biotinylated material. The overall workflow was quite straightforward, with 2 biological replicates used and comparing to wild-type parasites to determine proximal proteins. Spectral counts for proteins in wild-type parasites were simply subtracted from the spectral counts recorded for BirA*-KH expressing parasites. Ribosomal proteins were manually removed as likely contaminants, hits with a difference in spectral counts >10 were then considered proximal, although a basal body protein of potential interest was included despite not meeting the criteria. No statistical testing was performed, but to provide additional evidence of interactors a tandem affinity purification (TAP) experiment on KH was carried out. KH is associated with insoluble cytoskeletal structures, not an issue for proximity biotinylation but a challenge for an immunoprecipitation type approach where interactions generally need to be preserved during purification. To provide a route forward, the authors endogenously N-terminally tagged KH with the HBH tag. This tag contains a bacteria derived peptide that is biotinylated by endogenous biotin ligases in yeast and mammals, flanked by 6xHis, enabling TAP enrichment under denaturing conditions (Tagwerker et al., 2006). Parasites were cross-linked with formaldehyde to covalently link associating proteins, then lysed in 8M urea to solubilise complexes before denaturing TAP enrichment of KH associated complexes. Material was TMT labelled for a fully quantitative approach. Comparing the proximity biotinylation and TAP results revealed 3 proteins detected by both strategies, two of which were validated as novel KH associated proteins and demonstrated to be required for amastigote proliferation in macrophages.

Our group has recently completed two projects where we extended the use of proximity biotinylation to understanding fundamental aspects of chromatin biology in *Leishmania* (Geoghegan et al., 2021; Jones et al., 2021). We firstly discovered that we could increase the capture of proximal proteins by performing a limiting DSP cross-linking step after proximity biotinylation, an approach we termed XL-BioID (Geoghegan et al., 2021). This led to a ~10 fold increase in proximal protein signal intensity. We applied this technique to protein kinases belonging to the *Leishmania* kinetochore, a large complex that assembles on chromosomes, linking them to the mitotic spindle for segregation during mitosis. Kinetochore kinases KKT2, KKT3 and KKT19 were endogenously tagged with BirA* and used as baits in the XL-BioID experiment to explore the *Leishmania* kinetochore. We could detect 16 of the 25 predicted inner kinetochore proteins and discovered two novel kinetochore proteins, KKT24 and KKT26. Perhaps an underappreciated feature of proximity biotinylation is that the data it provides is not just a parts list for a complex of interest, rather it is also a snapshot of the dynamic cellular processes taking place in the vicinity of the bait protein. This aspect of proximity biotinylation data was apparent from our kinetochore study where we detected factors important for regulating chromatin structure, chromosome segregation factors and DNA replication factors proximal to kinetochore kinases. Interestingly, several nucleolar proteins were found in our proximity data, suggesting that the kinetochores associate with the nucleolus during the cell cycle which may be important for regulating their assembly.

To gain insight into assembly of the kinetochore during the cell cycle we used miniTurbo tagged KKT3, allowing us to take 30 minute biotinylation snapshots of the kinetochore in hydroxyurea synchronised parasites at ES (early-S), S and G2/M phases. Coverage of core kinetochore proteins was lower compared to using BirA*, for example at ES (early-S), 6 proximal KKTs were detected. Possible reasons for this could be due to different placement of the tag on the bait and faster labelling kinetics generating more noise. Nonetheless, we could follow changes in abundance amongst the detected proteins. For example, KKT4 was weakly proximal at ES, but became significantly more enriched during the cell cycle, reflecting its possible role in linking the kinetochore to the microtubule spindle, which takes place later in the cell cycle (Llauró et al., 2018).

Taking advantage of the increased capture of proximal proteins by XL-BioID in our investigation of the *Leishmania* kinetochore (Geoghegan et al., 2021), we developed proximity phosphoproteomics; enriching phosphopeptides from proximal proteins to add another dimension to proximity biotinylation data, namely the ability to track individual phosphosites at a complex of interest (Geoghegan et al., 2021). We quantified proximal phosphosites at the kinetochore as parasites progressed through the cell cycle. Label free quantification was used for both proximal proteins and phosphosites. Since the proximal phosphosites were low in abundance and likely to have higher variability, we used 5 biological replicates to give us sufficient statistical power to determine proximal phosphosites with limma, comparing intensities with a spatial reference. Limma is an R package for statistical analysis of various omics data, its application to proximity biotinylation data is discussed in more detail below (Ritchie et al., 2015). Our analysis revealed groups of phosphorylation sites with distinct dynamics, some changed in abundance in the same direction as the parent protein, but others showed highly dynamic behaviour during the cell cycle which was not a straightforward reflection of abundance of the protein at the kinetochore. These latter proximal phosphosites, such as KKT2 S493 and S530, may play important roles in directing assembly of the kinetochore. To discover proximal phosphorylation sites that may be part of kinase driven signalling pathways at the kinetochore, we also applied proximity phosphoproteomics in the presence of a covalent inhibitor of the kinetochore kinases CLK1 (KKT10)/CLK2 (KKT19). We were able to detect a small subset of proximal phosphorylation sites that decreased at G2/M after KKT10/KKT19 inhibition with AB1 (Saldivia et al., 2020), including 3 affected phosphosites on KKT2 which is a known substrate of these kinases. Thus proximity phosphoproteomics enabled us to not only quantify phosphorylation sites at the kinetochore during the cell cycle, but also to observe the effect of kinase inhibition on phosphorylation sites within this complex, providing important information to begin understanding individual kinase mediated signalling pathways.

In our experiments we used a spatial reference to assess the background, this greatly assisted in determining which proteins and phosphorylation sites were proximal to the kinetochore kinases. We chose a chromatin reader, BDF5, which we endogenously tagged with BirA* or miniTurbo. BDF5 was an ideal spatial reference for the kinetochore, as although both are chromatin associated, they localise to distinct chromatin domains, allowing accurate determination of off-target biotinylation. BDF5 was also a protein of interest in its own right, thus BDF5 and the kinetochore acted as reciprocal spatial references allowing us to extract maximum biological insight from mass spectrometry time.

BDF5 is a predicted acetyl-lysine histone reader which accumulates at transcription start sites and plays an essential role in regulating transcription in *Leishmania* (Jones et al., 2021). Our XL-BioID investigation revealed a core set of proximal proteins that included other bromodomain proteins BDF3, BDF4, a histone acetyltransferase HAT2, another predicted acetyl-lysine reader and several novel proteins. Evidence of a range of other chromatin/transcription associated processes occurring nearby was also captured by proximity biotinylation, such as detection of DNA replication and repair proteins, splicing factors, polyadenylation factors and RNA quality control proteins. Further analysis identified BDF5 as a regulator of transcription while proximal processes such as mRNA trans-splicing were unaffected. As with the kinetochore, we also discovered proximal phosphorylation sites which increased in number as parasites progressed through the cell cycle, although the overall number of phosphorylation sites detected was lower compared to the kinetochore.

## Generating Reliable Spatial Information: Experimental Design

In a proximity biotinylation experiment, direct interactors but also proximal proteins to the bait will become labelled. This is a distinct advantage of the method since it provides an insight into the molecular landscape surrounding a protein of interest. However, it means that a typical experiment may result in thousands of proteins being identified from the enriched biotinylated material. Determining which of these are proximal with high confidence will then depend on whether the experiment has been designed appropriately and also robust quantitative analysis of the mass spectrometry data. The overall aim when planning a proximity biotinylation experiment is to maximise the signal from proximal proteins whilst minimising background noise. Based on the examples in the literature we suggest careful consideration of the following points to increase the probability of a successful experiment:

1. Choice of enzyme. With an ever expanding repertoire of biotinylators to choose from, selecting the most suitable enzyme for the biological question may not be straightforward. A first consideration which might help narrow down the choice is the time scale that is being probed. If dynamic signalling networks are being investigated where a time resolution of minutes is required then an enzyme with fast kinetics such as APEX2 would be appropriate. If the goal is to characterize the steady state composition of a protein complex of interest, then BirA* with slower labelling kinetics may provide cleaner data for reasons outlined above. For intermediate cases, TurboID may be a useful enzyme, although the significant amount of biotinylation that can take place even before adding biotin would increase background noise. The related miniTurbo enzyme may be considered with caution, as it appears to be less stable in some contexts (May et al., 2020). The concentration of exogenous biotin should be tailored to the chosen biotin ligase, up to 500μM biotin is used for miniTurbo or TurboID and 50-150μM biotin for BirA* (Branon et al., 2018; Geoghegan et al., 2021; Zhou et al., 2018a).
2. Biotinylator location. Having selected an enzyme, the next decision is to determine where to place the biotinylator in the primary sequence of the bait protein. Factors to consider are common to the construction of any fusion protein. For N-terminal fusion, the sequence should be checked for the presence of any targeting signal that directs the proteins to a particular organelle such as the ER, since fusion to a biotinylator would likely disrupt targeting. Both termini should also be checked for domains known to be important for bait function. Ultimately though, the biotinylator-bait fusion protein should be expressed and correct localisation as well as function of the bait checked experimentally. Evidence that the biotinylator is active can be checked by streptavidin pull down from cultures supplemented or not with biotin, followed by western blotting to detect auto-biotinylation of the bait. Increased bait enrichment from biotin supplemented cultures, after normalising for input levels, indicates the biotinylator is active. To maximise coverage of the protein complex, it may be necessary to fuse the biotinylator to multiple components and possibly even using both N and C terminal fusions. This may also be helpful in identifying high confidence proximal proteins by analysing those proteins present in all datasets.
3. Expression level. Levels of the fusion protein should be as close to those of the endogenous protein as possible which may be achieved by endogenous tagging. It may be tempting to increase the signal to noise ratio by overexpressing the fusion protein, however this can lead to protein mis-localisation, generating a large amount of background biotinylated material which can obscure the true proximal proteins of interest and actually reduce signal to noise, as observed in a proximity biotinylation study of clathrin adaptor protein AP-1 comparing endogenous tagging with a biotinylator vs. transient overexpression (Stockhammer et al., 2021).
4. Spatial reference. This is probably the most important component of a proximity biotinylation experiment. The spatial reference consists of the biotinylating enzyme targeted to the same sub-cellular compartment as the biotinylator-bait fusion protein, providing a compartment specific background of incidentally biotinylated proteins against which enriched proteins from the bait sample can be compared (Lobingier et al., 2017). For example’ if the bait protein resides in the nucleus, the spatial reference could be the expression of the biotinylator containing a nuclear localisation signal. Choosing an appropriate spatial reference has been shown to be critical for distinguishing between the complex of interest and bystander proteins (Lobingier et al., 2017). An efficient experimental design would be to choose another protein of interest to which the biotinylator is fused (**Figure 2C**). Ideally this second protein would reside in the same compartment and at similar levels to the first bait, whilst being in a distinct complex. The two biotinylator-protein fusions could then act as reciprocal controls, making maximal use of experimental resources such as mass spectrometry time (Geoghegan et al., 2021; Jones et al., 2021).

Where resources permit, pilot proximity biotinylation experiments are an invaluable investment, enabling the above principles to be tested on the particular system of study. This could simply take the form of testing various biotinylator-bait fusion constructs for biotinylation activity by western blotting extracts with labelled streptavidin (e.g.HRP). Ideally though, pilot samples from biotinylator-bait fusions are analysed by mass spectrometry which will provide more detailed information on the signal to noise characteristics of the experiment. The bait protein should be one of the top identified hits after endogenously biotinylated proteins. If there is already some information on expected proximal proteins, the list should be checked for recall of these. Identification of a large number of chaperone proteins and other factors involved in protein folding may indicate that the cell has difficulty expressing and correctly processing the fusion protein (Banks et al., 2018).

## Generating Reliable Spatial Information: Data Analysis

Determining the proximal proteins from often quite complex datasets requires quantitative analysis of the enriched proteins. This firstly requires a number of biological replicates so that any enrichment observed against the control sample can be assessed relative to the variability of the measurement. At a minimum, 3 biological replicates should be analysed for each sample group, a higher number of replicates increases statistical power and may increase the sensitivity for detecting more transient proximal proteins. Semi-quantitative approaches to analysing proximity biotinylation data based on spectral counting have been successfully used (Lambert et al., 2015), but for optimal sensitivity a fully quantitative strategy should be considered which could be metabolic labelling (e.g. SILAC), chemical labelling (e.g. TMT) or label free quantitation.

In principle, detecting whether an identified protein is proximal is simply a matter of comparing its intensity in the bait samples to the intensity in the control samples, if it is significantly higher in the bait samples, it indicates the protein is proximal to the bait. There are a range of statistical methods for assessing whether enrichment is significant. The simplest approach is to carry out a pairwise statistical test, such as a t-test, between the intensities measured for a protein in the control and bait samples. This is performed protein-by-protein; therefore on a typical proximity biotinylation dataset, the statistical test is applied thousands of times and multiple testing correction must be applied to the p-values. While valid, this approach considers each protein in isolation, not in context of the quantitative information present in the entire dataset and therefore might lack sensitivity. Methods developed to analyse AP-MS data such as COMPASS, MiST and SAINTq (Sowa et al., 2009; Jäger et al., 2011; Teo et al., 2016) can instead be applied to proximity biotinylation data. In particular, SAINTq has emerged as a powerful probability modelling based tool for scoring interaction data. It can be used to score small and large scale proximity biotinylation studies and has the advantage of providing a False Discovery Rate (FDR) estimate for proximal proteins (Geoghegan et al., 2021).

Comparing intensities between samples and determining significant differences is a problem familiar to the microarray field. Several powerful statistical methods have been developed to analyse microarray data which can be leveraged to sensitively detect proximal proteins in proximity biotinylation data. The most well-known of these is limma, an R package originally developed for analysing microarray data and later adapted for RNA-seq data (Ritchie et al., 2015). Limma provides a solution to the problem of variance estimation when there are a low number of biological replicates. A typical proximity biotinylation experiment may consist of 3 biological replicates in a control group and 3 biological replicates in a bait group. If a simple t-test was performed on each measured protein to determine the significance of a difference between the two groups, the variance is estimated from this small number of biological replicates for each protein. The variance estimation will not be very accurate and after performing this test on thousands of proteins, some proteins will appear to be significantly different as they happen to have a low variability. Conversely, those proteins which happen to have higher variability would not be called as significantly different and so may be missed. The key innovation in limma is that the variance for each protein is estimated from the measured variability of *all* proteins in the experiment in a process called empirical Bayes. This results in shrinking of estimated variance for higher variability proteins and increasing estimated variance for lower variability proteins. The overall effect is to make determination of significant differences between samples less dependent on chance variability.

Proximity biotinylation data may be visualised by plotting enrichment against p-values in a volcano plot. However, we suggest a more effective visualisation is to plot enrichment and p-values radially, plotting only data for proteins enriched against the control (Geoghegan et al., 2021). The bait protein normally has the lowest p-value and is therefore the central point in the plot, proximal proteins are then radially arranged close to the bait. Compared to volcano plots, this provides a better spread of proximal proteins which are the data of interest.

## Anticipated Improvements and Future Applications

Parasitologists were amongst the first to recognise and apply the power of proximity biotinylation to explore protein complexes and cellular processes, but it is still a relatively new technique under very active development. New biotin ligases will continue to be developed, likely drawing from sequences in metagenomic databases and using directed evolution to engineer desired characteristics such as fast labelling, small size, rapid folding, stability and possibly even a controllable labelling radius (Ke et al., 2021). Many biological systems including kinetoplastids express abundant endogenously biotinylated proteins which are unfortunately co-purified with proximity biotinylated proteins but are not of interest. Therefore a useful development would be a non-biotin proximity labelling system which would include a corresponding high affinity binder to enable specific purification of proximal material. Non-biotin proximity labelling methodologies do exist but usage is restricted due to non-diffusible labelling (PUP-IT) or requirement for a chemical catalyst (MicroMap) (Liu et al., 2018; Geri et al., 2020).

Proteins that form discrete structures have been the focus of most proximity biotinylation studies in kinetoplastids, but moving forward there are many interesting targets that do not form stable long-lived complexes and may have more diffuse localisations within the parasite. For example, these proteins may be part of signalling pathways that report on the parasite microenvironment in the host and instruct the parasite to differentiate. Conditional proximity biotinylation could be applied to proteins that have multiple localisations in the parasite, using recently developed split biotin ligase systems such as split TurboID (Cho et al., 2020). The bait would be fused to one half of the biotin ligase and another protein in the complex of interest is fused to the complementing biotin ligase portion. Close association between the two proteins re-constitutes biotin ligase activity so that only proximal proteins at the complex of interest are biotinylated. In theory this improves spatial specificity, but may also reduce signal as split enzymes often possess lower activity, it also requires knowledge of another interacting partner.

Another refinement is functional proximity biotinylation, where proximity material is enriched for proteins having certain biochemical characteristics or functions (Qin et al., 2021b). We demonstrated this in our proximity phosphoproteomics work on the *Leishmania* kinetochore. But proximal proteins bearing other PTMs could be enriched, such as glycosylation, acetylation, ubiquitin or SUMO. This interrogates PTMs within a complex of interest and is therefore a spatially focussed view of those modifications likely to be functionally important, as compared to many large scale PTM proteomics studies which are averages across whole cells or tissues. Finally, no studies have yet used proximity biotinylation to explore non-protein proximal biomolecules in kinetoplastids. Differential transcriptional regulation is largely absent in kinetoplastids, instead RNA binding proteins are the key regulators of mRNA levels (De Pablos et al., 2016). Exploring how RNA binding proteins modulate levels of certain transcripts could be possible by using APEX2, which can biotinylate proximal RNAs that can then be enriched and sequenced (Fazal et al., 2019). Non-biotinylation based labelling systems such as Cap-seq and Halo-seq could also be applied to investigate proximal RNAs (Wang et al., 2019; Engel et al., 2021). A recently introduced proximity labelling method, MicroMap, uses carbene based labelling (Geri et al., 2020). Carbenes are extremely reactive with a half life in the nanosecond range and were demonstrated to provide very spatially refined proximity labelling on the cell plasma membrane, they also react with C-H bonds and so could potentially label a very wide range of biomolecules such as RNA, DNA and lipids.

The intersection between chemistry and protein engineering will continue to expand the range of proximity labelling methods, offering kinetoplastid researchers a rich array of techniques with which to drive discovery in these unique parasites.

## Author Contributions

VG and NGJ wrote the manuscript with editing contributions from JCM. All authors contributed to the article and approved the submitted version.

## Funding

This work was funded by the Wellcome Trust (200807/Z/16/Z) and MRC GCRF (MR/P027989/1).

## Conflict of Interest

The authors declare that the research was conducted in the absence of any commercial or financial relationships that could be construed as a potential conflict of interest.

## Publisher’s Note

All claims expressed in this article are solely those of the authors and do not necessarily represent those of their affiliated organizations, or those of the publisher, the editors and the reviewers. Any product that may be evaluated in this article, or claim that may be made by its manufacturer, is not guaranteed or endorsed by the publisher.

## References

[B1] Akiyoshi B. Gull K. (2014). Discovery of Unconventional Kinetochores in Kinetoplastids. Cell 156, 1247–1258. doi: 10.1016/j.cell.2014.01.049 24582333 PMC3978658

[B2] An T. Zhou Q. Hu H. Cormaty H. Li Z. (2020). FAZ27 Cooperates With FLAM3 and ClpGM6 to Maintain Cell Morphology in Trypanosoma Brucei. J. Cell Sci. 133, 1–14. doi: 10.1242/jcs.245258 PMC7295586 32393602

[B3] Banks C. A. S. Miah S. Adams M. K. Eubanks C. G. Thornton J. L. Florens L.. (2018). Differential HDAC1/2 Network Analysis Reveals a Role for Prefoldin/CCT in HDAC1/2 Complex Assembly. Sci. Rep. 8, 13712. doi: 10.1038/s41598-018-32009-w 30209338 PMC6135828

[B4] Bar D. Z. Atkatsh K. Tavarez U. Erdos M. R. Gruenbaum Y. Collins F. S. (2018). Biotinylation by Antibody Recognition-a Method for Proximity Labeling. Nat. Methods 15, 127–133. doi: 10.1038/nmeth.4533 29256494 PMC5790613

[B5] Baudouin H. C. M. Pfeiffer L. Ochsenreiter T. (2020). A Comparison of Three Approaches for the Discovery of Novel Tripartite Attachment Complex Proteins in Trypanosoma Brucei. PloS Negl. Trop. Dis. 14, e0008568. doi: 10.1371/journal.pntd.0008568 32936798 PMC7521757

[B6] Branon T. C. Bosch J. A. Sanchez A. D. Udeshi N. D. Svinkina T. Carr S. A.. (2018). Efficient Proximity Labeling in Living Cells and Organisms With TurboID. Nat. Biotechnol. 36, 880–887. doi: 10.1038/nbt.4201 30125270 PMC6126969

[B7] Branon T. Han S. Ting A. (2017). Beyond Immunoprecipitation: Exploring New Interaction Spaces With Proximity Biotinylation. Biochemistry 56, 3297–3298. doi: 10.1021/acs.biochem.7b00466 28574262

[B8] Burza S. Croft S. L. Boelaert M. (2018). Leishmaniasis. Lancet 392, 951–970. doi: 10.1016/S0140-6736(18)31204-2 30126638

[B9] Cho K. F. Branon T. C. Rajeev S. Svinkina T. Udeshi N. D. Thoudam T.. (2020). Split-TurboID Enables Contact-Dependent Proximity Labeling in Cells. Proc. Natl. Acad. Sci. U. S. A. 117, 12143–12154. doi: 10.1073/pnas.1919528117 32424107 PMC7275672

[B10] Choi-Rhee E. Schulman H. Cronan J. E. (2004). Promiscuous Protein Biotinylation by Escherichia Coli Biotin Protein Ligase. Protein Sci. 13, 3043–3050. doi: 10.1110/ps.04911804 15459338 PMC2286582

[B11] Clayton C. E. (2016). Gene Expression in Kinetoplastids. Curr. Opin. Microbiol. 32, 46–51. doi: 10.1016/j.mib.2016.04.018 27177350

[B12] Dang H. Q. Zhou Q. Rowlett V. W. Hu H. Lee K. J. Margolin W.. (2017). Proximity Interactions Among Basal Body Components in Trypanosoma Brucei Identify Novel Regulators of Basal Body Biogenesis and Inheritance. MBio 8, 1–15. doi: 10.1128/mBio.02120-16 PMC5210500 28049148

[B13] De Pablos L. M. Ferreira T. R. Walrad P. B. (2016). Developmental Differentiation in Leishmania Lifecycle Progression: Post-Transcriptional Control Conducts the Orchestra. Curr. Opin. Microbiol. 34, 82–89. doi: 10.1016/j.mib.2016.08.004 27565628

[B14] De Pablos L. M. Kelly S. de Freitas Nascimento J. Sunter J. Carrington M. (2017). Characterization of RBP9 and RBP10, Two Developmentally Regulated RNA-Binding Proteins in Trypanosoma Brucei. Open Biol. 7, 1–14. doi: 10.1098/rsob.160159 PMC5413900 28381627

[B15] Dong X. Lim T. K. Lin Q. He C. Y. (2020). Basal Body Protein TbSAF1 Is Required for Microtubule Quartet Anchorage to the Basal Bodies in Trypanosoma Brucei. MBio 11, 1–19. doi: 10.1128/mBio.00668-20 PMC7291619 32518185

[B16] Engel K. L. Lo H.-Y. G. Goering R. Li Y. Spitale R. C. Taliaferro J. M. (2021). Analysis of Subcellular Transcriptomes by RNA Proximity Labeling With Halo-Seq. Nucleic Acids Res. 50, 1–17. doi: 10.1093/nar/gkab1185 PMC8887463 34875090

[B17] Erickson H. P. (2009). Size and Shape of Protein Molecules at the Nanometer Level Determined by Sedimentation, Gel Filtration, and Electron Microscopy. Biol. Proced. Online 11, 32–51. doi: 10.1007/s12575-009-9008-x 19495910 PMC3055910

[B18] Fazal F. M. Han S. Parker K. R. Kaewsapsak P. Xu J. Boettiger A. N.. (2019). Atlas of Subcellular RNA Localization Revealed by APEX-Seq. Cell 178, 473–490.e26. doi: 10.1016/j.cell.2019.05.027 31230715 PMC6786773

[B19] Geoghegan V. Jones N. G. Dowle A. Mottram J. C. (2021). Protein Kinase Signalling at the Leishmania Kinetochore Captured by XL-BioID. bioRxiv 2021.07.08.451598. doi: 10.1101/2021.07.08.451598

[B20] Geri J. B. Oakley J. V. Reyes-Robles T. Wang T. McCarver S. J. White C. H.. (2020). Microenvironment Mapping *via* Dexter Energy Transfer on Immune Cells. Science 367, 1091–1097. doi: 10.1126/science.aay4106 32139536 PMC7336666

[B21] Gingras A.-C. Abe K. T. Raught B. (2019). Getting to Know the Neighborhood: Using Proximity-Dependent Biotinylation to Characterize Protein Complexes and Map Organelles. Curr. Opin. Chem. Biol. 48, 44–54. doi: 10.1016/j.cbpa.2018.10.017 30458335

[B22] Gingras A.-C. Gstaiger M. Raught B. Aebersold R. (2007). Analysis of Protein Complexes Using Mass Spectrometry. Nat. Rev. Mol. Cell Biol. 8, 645–654. doi: 10.1038/nrm2208 17593931

[B23] Hilton N. A. Sladewski T. E. Perry J. A. Pataki Z. Sinclair-Davis A. N. Muniz R. S.. (2018). Identification of TOEFAZ1-Interacting Proteins Reveals Key Regulators of Trypanosoma Brucei Cytokinesis. Mol. Microbiol. 109, 306–326. doi: 10.1111/mmi.13986 29781112 PMC6359937

[B24] Hopkins C. Gibson A. Stinchcombe J. Futter C. (2000). Chimeric Molecules Employing Horseradish Peroxidase as Reporter Enzyme for Protein Localization in the Electron Microscope. Methods Enzymol. 327, 35–45. doi: 10.1016/S0076-6879(00)27265-0 11044972

[B25] Hu H. An T. Kurasawa Y. Zhou Q. Li Z. (2019). The Trypanosome-Specific Proteins FPRC and CIF4 Regulate Cytokinesis Initiation by Recruiting CIF1 to the Cytokinesis Initiation Site. J. Biol. Chem. 294, 16672–16683. doi: 10.1074/jbc.RA119.010538 31540971 PMC6851298

[B26] Hu H. Zhou Q. Li Z. (2015). SAS-4 Protein in Trypanosoma Brucei Controls Life Cycle Transitions by Modulating the Length of the Flagellum Attachment Zone Filament. J. Biol. Chem. 290, 30453–30463. doi: 10.1074/jbc.M115.694109 26504079 PMC4683267

[B27] Ishihama Y. Oda Y. Tabata T. Sato T. Nagasu T. Rappsilber J.. (2005). Exponentially Modified Protein Abundance Index (emPAI) for Estimation of Absolute Protein Amount in Proteomics by the Number of Sequenced Peptides Per Protein. Mol. Cell. Proteomics 4, 1265–1272. doi: 10.1074/mcp.M500061-MCP200 15958392

[B28] Jäger S. Cimermancic P. Gulbahce N. Johnson J. R. McGovern K. E. Clarke S. C.. (2011). Global Landscape of HIV–human Protein Complexes. Nature 481, 365–370. doi: 10.1038/nature10719 22190034 PMC3310911

[B29] Jones N. G. Geoghegan V. Moore G. Carnielli J. B. T. Newling K. Calderón F.. (2021). Bromodomain Factor 5 is an Essential Transcriptional Regulator of the Leishmania Genome. bioRxiv 2021.09.29.462384. doi: 10.1101/2021.09.29.462384

[B30] Kelly F. D. Tran K. D. Hatfield J. Schmidt K. Sanchez M. A. Landfear S. M. (2020). A Cytoskeletal Protein Complex is Essential for Division of Intracellular Amastigotes of Leishmania Mexicana. J. Biol. Chem. 295, 13106–13122. doi: 10.1074/jbc.RA120.014065 32719012 PMC7489907

[B31] Ke M. Yuan X. He A. Yu P. Chen W. Shi Y.. (2021). Spatiotemporal Profiling of Cytosolic Signaling Complexes in Living Cells by Selective Proximity Proteomics. Nat. Commun. 12, 71. doi: 10.1038/s41467-020-20367-x 33397984 PMC7782698

[B32] Kido K. Yamanaka S. Nakano S. Motani K. Shinohara S. Nozawa A.. (2020). AirID, a Novel Proximity Biotinylation Enzyme, for Analysis of Protein–Protein Interactions. Elife 9, e54983. doi: 10.7554/eLife.54983.sa2 32391793 PMC7302878

[B33] Kim D. I. Birendra K. C. Zhu W. Motamedchaboki K. Doye V. Roux K. J. (2014). Probing Nuclear Pore Complex Architecture With Proximity-Dependent Biotinylation. Proc. Natl. Acad. Sci. U. S. A. 111, E2453–E2461. doi: 10.1073/pnas.1406459111 24927568 PMC4066523

[B34] Kim D. I. Jensen S. C. Noble K. A. Kc B. Roux K. H. Motamedchaboki K.. (2016). An Improved Smaller Biotin Ligase for BioID Proximity Labeling. Mol. Biol. Cell 27, 1188–1196. doi: 10.1091/mbc.E15-12-0844 26912792 PMC4831873

[B35] Kostygov A. Y. Karnkowska A. Votýpka J. Tashyreva D. Maciszewski K. Yurchenko V.. (2021). Euglenozoa: Taxonomy, Diversity and Ecology, Symbioses and Viruses. Open Biol. 11, 200407. doi: 10.1098/rsob.200407 33715388 PMC8061765

[B36] Kwon K. Beckett D. (2000). Function of a Conserved Sequence Motif in Biotin Holoenzyme Synthetases. Protein Sci. 9, 1530–1539. doi: 10.1110/ps.9.8.1530 10975574 PMC2144715

[B37] Lambert J.-P. Tucholska M. Go C. Knight J. D. R. Gingras A.-C. (2015). Proximity Biotinylation and Affinity Purification are Complementary Approaches for the Interactome Mapping of Chromatin-Associated Protein Complexes. J. Proteomics 118, 81–94. doi: 10.1016/j.jprot.2014.09.011 25281560 PMC4383713

[B38] Lam S. S. Martell J. D. Kamer K. J. Deerinck T. J. Ellisman M. H. Mootha V. K.. (2015). Directed Evolution of APEX2 for Electron Microscopy and Proximity Labeling. Nat. Methods 12, 51–54. doi: 10.1038/nmeth.3179 25419960 PMC4296904

[B39] Libisch M. G. Rego N. Robello C. (2021). Transcriptional Studies on Trypanosoma Cruzi – Host Cell Interactions: A Complex Puzzle of Variables. Front. Cell. Infect. Microbiol. 11. doi: 10.3389/fcimb.2021.692134 PMC8248493 34222052

[B40] Liu Q. Zheng J. Sun W. Huo Y. Zhang L. Hao P.. (2018). A Proximity-Tagging System to Identify Membrane Protein-Protein Interactions. Nat. Methods 15, 715–722. doi: 10.1038/s41592-018-0100-5 30104635

[B41] Llauró A. Hayashi H. Bailey M. E. Wilson A. Ludzia P. Asbury C. L.. (2018). The Kinetoplastid Kinetochore Protein KKT4 is an Unconventional Microtubule Tip–Coupling Protein. J. Cell Biol. 217, 3886–3900. doi: 10.1083/jcb.201711181 30209069 PMC6219724

[B42] Lobingier B. T. Hüttenhain R. Eichel K. Miller K. B. Ting A. Y. von Zastrow M.. (2017). An Approach to Spatiotemporally Resolve Protein Interaction Networks in Living Cells. Cell 169, 350–360.e12. doi: 10.1016/j.cell.2017.03.022 28388416 PMC5616215

[B43] May D. G. Scott K. L. Campos A. R. Roux K. J. (2020). Comparative Application of BioID and TurboID for Protein-Proximity Biotinylation. Cells 9, 1–20. doi: 10.3390/cells9051070 PMC7290721 32344865

[B44] McAllaster M. R. Ikeda K. N. Lozano-Núñez A. Anrather D. Unterwurzacher V. Gossenreiter T.. (2015). Proteomic Identification of Novel Cytoskeletal Proteins Associated With TbPLK, an Essential Regulator of Cell Morphogenesis in Trypanosoma Brucei. Mol. Biol. Cell 26, 3013–3029. doi: 10.1091/mbc.E15-04-0219 26133384 PMC4551316

[B45] Mehla J. Caufield J. H. Sakhawalkar N. Uetz P. (2017). A Comparison of Two-Hybrid Approaches for Detecting Protein-Protein Interactions. Methods Enzymol. 586, 333–358. doi: 10.1016/bs.mie.2016.10.020 28137570 PMC5737774

[B46] Miyagawa-Yamaguchi A. Kotani N. Honke K. (2014). Segregation of Lipid Rafts Revealed by the EMARS Method Using GPI-Anchored HRP Fusion Proteins. Trends Glycosci. Glycotechnol. 26, 59–69. doi: 10.4052/tigg.26.59

[B47] Morriswood B. Havlicek K. Demmel L. Yavuz S. Sealey-Cardona M. Vidilaseris K.. (2013). Novel Bilobe Components in Trypanosoma Brucei Identified Using Proximity-Dependent Biotinylation. Eukaryot. Cell 12, 356–367. doi: 10.1128/EC.00326-12 23264645 PMC3571296

[B48] Paek J. Kalocsay M. Staus D. P. Wingler L. Pascolutti R. Paulo J. A.. (2017). Multidimensional Tracking of GPCR Signaling *via* Peroxidase-Catalyzed Proximity Labeling. Cell 169, 338–349.e11. doi: 10.1016/j.cell.2017.03.028 28388415 PMC5514552

[B49] Pandey M. Huang Y. Lim T. K. Lin Q. He C. Y. (2020). Flagellar Targeting of an Arginine Kinase Requires a Conserved Lipidated Protein Intraflagellar Transport (LIFT) Pathway in Trypanosoma Brucei. J. Biol. Chem. 295, 11326–11336. doi: 10.1074/jbc.RA120.014287 32587088 PMC7415996

[B50] Pays E. Nolan D. P. (2021). Genetic and Immunological Basis of Human African Trypanosomiasis. Curr. Opin. Immunol. 72, 13–20. doi: 10.1016/j.coi.2021.02.007 33721725 PMC8589022

[B51] Povelones M. L. (2014). Beyond Replication: Division and Segregation of Mitochondrial DNA in Kinetoplastids. Mol. Biochem. Parasitol. 196, 53–60. doi: 10.1016/j.molbiopara.2014.03.008 24704441

[B52] Pyrih J. Rašková V. Škodová-Sveráková I. Pánek T. Lukeš J. (2020). ZapE/Afg1 Interacts With Oxa1 and its Depletion Causes a Multifaceted Phenotype. PloS One 15, e0234918. doi: 10.1371/journal.pone.0234918 32579605 PMC7314023

[B53] Qin W. Cho K. F. Cavanagh P. E. Ting A. Y. (2021a). Deciphering Molecular Interactions by Proximity Labeling. Nat. Methods 18, 133–143. doi: 10.1038/s41592-020-01010-5 33432242 PMC10548357

[B54] Qin W. Myers S. A. Carey D. K. Carr S. A. Ting A. Y. (2021b). Spatiotemporally-Resolved Mapping of RNA Binding Proteins via Functional Proximity Labeling Reveals a Mitochondrial mRNA Anchor Promoting Stress Recovery. Nat. Commun. 12, 4980. doi: 10.1038/s41467-021-25259-2 34404792 PMC8370977

[B55] Ramanathan M. Majzoub K. Rao D. S. Neela P. H. Zarnegar B. J. Mondal S.. (2018). RNA-Protein Interaction Detection in Living Cells. Nat. Methods 15, 207–212. doi: 10.1038/nmeth.4601 29400715 PMC5886736

[B56] Remnant L. Booth D. G. Vargiu G. Spanos C. Kerr A. R. W. Earnshaw W. C. (2019). *In Vitro* BioID: Mapping the CENP-A Microenvironment With High Temporal and Spatial Resolution. Mol. Biol. Cell 30, 1314–1325. doi: 10.1091/mbc.E18-12-0799 30892990 PMC6724601

[B57] Ritchie M. E. Phipson B. Wu D. Hu Y. Law C. W. Shi W.. (2015). Limma Powers Differential Expression Analyses for RNA-Sequencing and Microarray Studies. Nucleic Acids Res. 43, e47. doi: 10.1093/nar/gkv007 25605792 PMC4402510

[B58] Roux K. J. Kim D. I. Raida M. Burke B. (2012). A Promiscuous Biotin Ligase Fusion Protein Identifies Proximal and Interacting Proteins in Mammalian Cells. J. Cell Biol. 196, 801–810. doi: 10.1083/jcb.201112098 22412018 PMC3308701

[B59] Saldivia M. Fang E. Ma X. Myburgh E. Carnielli J. B. T. Bower-Lepts C.. (2020). Targeting the Trypanosome Kinetochore With CLK1 Protein Kinase Inhibitors. Nat. Microbiol. 5, 1207–1216. doi: 10.1038/s41564-020-0745-6 32661312 PMC7610364

[B60] Samavarchi-Tehrani P. Samson R. Gingras A.-C. (2020). Proximity Dependent Biotinylation: Key Enzymes and Adaptation to Proteomics Approaches. Mol. Cell. Proteomics 19, 757–773. doi: 10.1074/mcp.R120.001941 32127388 PMC7196579

[B61] Sowa M. E. Bennett E. J. Gygi S. P. Harper J. W. (2009). Defining the Human Deubiquitinating Enzyme Interaction Landscape. Cell 138, 389–403. doi: 10.1016/j.cell.2009.04.042 19615732 PMC2716422

[B62] Stockhammer A. Benz L. S. Freund C. Kuropka B. Bottanelli F. (2021). When Less is More - Endogenous Tagging With TurboID Increases the Sensitivity of Proximity Labelling-Based Experiments. bioRxiv 2021.11.19.469212. doi: 10.1101/2021.11.19.469212

[B63] Tagwerker C. Flick K. Cui M. Guerrero C. Dou Y. Auer B.. (2006). A Tandem Affinity Tag for Two-Step Purification Under Fully Denaturing Conditions: Application in Ubiquitin Profiling and Protein Complex Identification Combined With *In Vivo*cross-Linking. Mol. Cell. Proteomics 5, 737–748. doi: 10.1074/mcp.M500368-MCP200 16432255

[B64] Teo G. Koh H. Fermin D. Lambert J.-P. Knight J. D. R. Gingras A.-C.. (2016). SAINTq: Scoring Protein-Protein Interactions in Affinity Purification - Mass Spectrometry Experiments With Fragment or Peptide Intensity Data. Proteomics 16, 2238–2245. doi: 10.1002/pmic.201500499 27119218

[B65] Tiengwe C. Marcello L. Farr H. Dickens N. Kelly S. Swiderski M.. (2012). Genome-Wide Analysis Reveals Extensive Functional Interaction Between DNA Replication Initiation and Transcription in the Genome of Trypanosoma Brucei. Cell Rep. 2, 185–197. doi: 10.1016/j.celrep.2012.06.007 22840408 PMC3607257

[B66] Vélez-Ramírez D. E. Shimogawa M. M. Ray S. S. Lopez A. Rayatpisheh S. Langousis G.. (2021). APEX2 Proximity Proteomics Resolves Flagellum Subdomains and Identifies Flagellum Tip-Specific Proteins in Trypanosoma Brucei. mSphere 6, 1–19. doi: 10.1128/mSphere.01090-20 PMC8141408 33568455

[B67] Wang P. Tang W. Li Z. Zou Z. Zhou Y. Li R.. (2019). Mapping Spatial Transcriptome With Light-Activated Proximity-Dependent RNA Labeling. Nat. Chem. Biol. 15, 1110–1119. doi: 10.1038/s41589-019-0368-5 31591565

[B68] Wartlick O. Kicheva A. González-Gaitán M. (2009). Morphogen Gradient Formation. Cold Spring Harb. Perspect. Biol. 1, a001255. doi: 10.1101/cshperspect.a001255 20066104 PMC2773637

[B69] Zhao X. Bitsch S. Kubitz L. Schmitt K. Deweid L. Roehrig A.. (2021). ultraID: A Compact and Efficient Enzyme for Proximity-Dependent Biotinylation in Living Cells. bioRxiv 2021.06.16.448656. doi: 10.1101/2021.06.16.448656 PMC9253107 35788163

[B70] Zhou Q. An T. Pham K. T. M. Hu H. Li Z. (2018a). The CIF1 Protein is a Master Orchestrator of Trypanosome Cytokinesis That Recruits Several Cytokinesis Regulators to the Cytokinesis Initiation Site. J. Biol. Chem. 293, 16177–16192. doi: 10.1074/jbc.RA118.004888 30171070 PMC6200942

[B71] Zhou Q. Hu H. Li Z. (2016). An EF-Hand-Containing Protein in Trypanosoma Brucei Regulates Cytokinesis Initiation by Maintaining the Stability of the Cytokinesis Initiation Factor Cif1. J. Biol. Chem. 291, 14395–14409. doi: 10.1074/jbc.M116.726133 27226595 PMC4938165

[B72] Zhou Q. Lee K. J. Kurasawa Y. Hu H. An T. Li Z. (2018b). Faithful Chromosome Segregation in Trypanosoma Brucei Requires a Cohort of Divergent Spindle-Associated Proteins With Distinct Functions. Nucleic Acids Res. 46, 8216–8231. doi: 10.1093/nar/gky557 29931198 PMC6144804

